# Loss of Arf4 causes severe degeneration of the exocrine pancreas but not cystic kidney disease or retinal degeneration

**DOI:** 10.1371/journal.pgen.1006740

**Published:** 2017-04-14

**Authors:** Jillian N. Pearring, Jovenal T. San Agustin, Ekaterina S. Lobanova, Christopher J. Gabriel, Eric C. Lieu, William J. Monis, Michael W. Stuck, Lara Strittmatter, Samer M. Jaber, Vadim Y. Arshavsky, Gregory J. Pazour

**Affiliations:** 1 Department of Ophthalmology, Duke University School of Medicine, Durham, North Carolina, United States of America; 2 Program in Molecular Medicine, University of Massachusetts Medical School, Worcester, Massachusetts, United States of America; 3 Electron Microscopy Core, University of Massachusetts Medical School, Worcester, Massachusetts, United States of America; 4 Department of Animal Medicine, University of Massachusetts Medical School, Worcester, Massachusetts, United States of America; Washington University School of Medicine, UNITED STATES

## Abstract

Arf4 is proposed to be a critical regulator of membrane protein trafficking in early secretory pathway. More recently, Arf4 was also implicated in regulating ciliary trafficking, however, this has not been comprehensively tested *in vivo*. To directly address Arf4’s role in ciliary transport, we deleted *Arf4* specifically in either rod photoreceptor cells, kidney, or globally during the early postnatal period. *Arf4* deletion in photoreceptors did not cause protein mislocalization or retinal degeneration, as expected if Arf4 played a role in protein transport to the ciliary outer segment. Likewise, Arf4 deletion in kidney did not cause cystic disease, as expected if Arf4 were involved in general ciliary trafficking. In contrast, global Arf4 deletion in the early postnatal period resulted in growth restriction, severe pancreatic degeneration and early death. These findings are consistent with Arf4 playing a critical role in endomembrane trafficking, particularly in the pancreas, but not in ciliary function.

## Author summary

Primary cilia are sensory organelles found on most cells and contain specific receptors that detect extracellular stimuli. Defects in trafficking receptors to cilia cause a diverse set of diseases called ciliopathies, which include polycystic kidney disease, obesity, cerebral anomalies and retinal degeneration. Based mostly on *in vitro* studies, the small GTPase Arf4 was thought to be critically important for localizing rhodopsin to the outer segment of photoreceptor cells and cystoproteins to kidney cilia. Here we genetically remove Arf4 from mice in either a tissue specific or time dependent manner. To our surprise, the loss of Arf4 does not cause retinal degeneration or cystic kidney disease. Since ciliary dysfunction causes retinal degeneration and cystic disease, our findings indicate that Arf4 does not play a role in ciliary function. Instead, mice have zymogen granule defects and degeneration of the exocrine pancreas supporting roles for Arf4 in regulating endomembrane trafficking in specific cells.

## Introduction

Primary cilia are microtubule-based organelles, which perform sensory functions important for health and development in vertebrates. Severe defects in primary cilia lead to embryonic lethality. Mild defects cause a wide range of syndromic diseases, termed ciliopathies, which manifest as a spectrum of features including obesity, retinal degeneration, cerebral anomalies and renal disease [1]. Each cilium contains a set of specific proteins, some shared across cell types and others adapted to perform unique cell-specific functions. This specialization relies on robust intracellular trafficking mechanisms whose malfunctions underlie a variety of pathological conditions [2, 3].

These mechanisms remain a subject of active investigation, with many proteins being delivered into the cilium through intraflagellar transport (IFT) [4–7]. Less is known about the molecular players responsible for designating proteins for ciliary trafficking upon their exit from the biosynthetic membranes. Many ciliary proteins contain short sequences used for their specific targeting [8, 9]. One of the better studied cases is the visual pigment rhodopsin, which contains a four amino acid (V[S/A]PA) targeting sequence at its C-terminus known as the VXPX motif [10, 11]. Human patients containing mutations in either the V or P residues exhibit autosomal dominant retinitis pigmentosa [12], and deletion of these residues in mice results in rhodopsin mislocalization followed by photoreceptor cell death [13–15]. These residues were also found to be important in an *in vitro* assay for the formation of rhodopsin carrier vesicles in the Golgi [16]. A similar RVXP motif is present in other ciliary-localized proteins such as the CNGB1b subunit of the olfactory CNG channel, polycystin-1, polycystin-2 and presenlin-2 [17–20]. The P-to-A replacement in the VXPX motif of presenilin-2 was also shown to mislocalize this protein from the basal body of epidermal suprabasal cells [18].

The work by Deretic and colleagues suggested that the C-terminal VXPX sequence in rhodopsin is recognized by Arf4 [21]. Arf4 is a small GTPase regulating protein trafficking in the early secretory pathway. It is typically localized in the ER/Golgi intermediate compartment and cis-Golgi [22, 23]. Deretic and colleagues hypothesized that, in addition to this well-established function, Arf4 directs ciliary trafficking of rhodopsin in trans-Golgi. Most importantly, they showed that Arf4 interacts with rhodopsin’s cytoplasmic C-terminus *in vitro* [21] and that expression of a dominant-negative Arf4 mutant in frog rods causes a partial rhodopsin mislocalization from the cilium [16]. Subsequent work suggested that Arf4 is required for trafficking other ciliary proteins, including polycystins and fibrocystin [17, 24]. These findings implicated Arf4 as a key player in sorting transmembrane proteins to the cilium, suggesting that its malfunction or loss would lead to human diseases such as retinal degeneration and polycystic kidney disease.

To address the functional role of Arf4 *in vivo*, an *Arf4* knockout mouse was generated, but this mutation resulted in embryonic lethality between days 9 and 10 [24]. Although embryonic lethality occurs in mice with severe defects in ciliary assembly, the embryonic node of the *Arf4* knockout mouse had normal cilia, which were functional since all embryos broke left-right symmetry properly and formed a D-looped heart. In wild type mice, *Arf4* is highly expressed in the visceral endoderm starting at embryonic day 7. In *Arf4* mutants, the microvilli and lysosomes of the visceral endoderm cells were disrupted and the localization of the endocytic receptor megalin was altered. Since the visceral endoderm is the major secretory and absorptive tissue of the developing embryo it is likely that these defects are the cause of lethality in the *Arf4* knockout mice. This is consistent with the established function of Arf4 in mediating ER/Golgi protein trafficking, but does not imply a defect in ciliogenesis as no cilia were detected on these cells.

One approach to circumvent the embryonic lethality of germline *Arf4* knockout, reported in a recent study [18], was to knockdown Arf4 using a lentiviral shRNA infection at E9.5. The authors investigated the skin phenotype of these animals and observed presenilin-2 mislocalization from the basal body of the epidermal cells, defects in Notch signaling, and polydactyly. Here, we used a more straightforward approach to assess the role of Arf4 in adult tissues by generating a floxed *Arf4* mouse. To our surprise, Arf4 knockout from photoreceptors did not affect rhodopsin localization or photoreceptor morphology, and Arf4 knockout from kidney did not affect ciliogenesis or cause cystic disease. In contrast, the Arf4 knockout caused severe degeneration of the exocrine pancreas, consistent with Arf4 playing a critical role in endomembrane trafficking but not ciliary function.

## Results

### Characterization of the tamoxifen-inducible Arf4 knockout mouse

**Fig 1:**
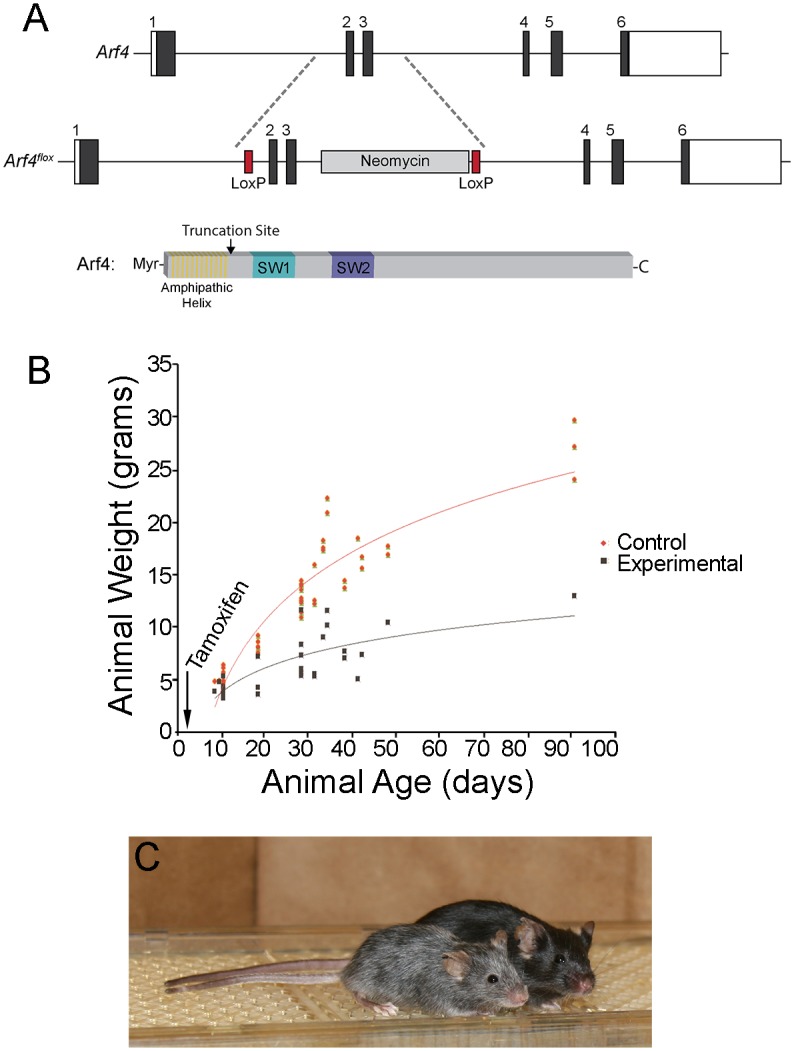
Generation of the floxed Arf4 allele to characterize tamoxifen-induced deletion of *Arf4* postnatally. A. The mouse *Arf4* gene consists of six exons. The *Arf4*^*flox*^ mouse has LoxP sites flanking exons 2 and 3. Upon Cre recombinase expression, a frame shift is introduced after residue 22 causing early truncation of this 180 amino acid protein. The position of the early truncation site in relationship to the N-terminal functional regions of ARF4 is illustrated on the cartoon at the bottom of this panel. B. Tamoxifen was administered to *Arf4*^*flox*^*/CagCreER* mice by intraperitoneal injection on P2. Animals were genotyped at P10 where only 2/3 as many experimental mice were identified as expected (P = 0.0002 by Chi-square analysis). Orange and black dots represent weights of control and experimental mice at harvest. Curves are logarithmic best fits and are significantly different (P = 0.0003 by F test). C. At P91, Arf4 experimental mice are smaller in size and have grey coat color compared to littermate controls. Both experimental and control mice were black until 6–7 weeks of age when the experimental mice turned grey. Experimental mice were noticeably smaller and died at random times.

To generate a conditional Arf4 knockout mouse (*Arf4*^*flox*^), we floxed exons 2 and 3 that encode the functionally indispensable switch 1 and switch 2 regions of Arf4. Cre recombination results in a frame shift after residue 22 and early termination 6 residues downstream from this residue (Fig 1A). While this peptide cannot retain the function of a small GTPase, N-termini of Arf proteins have been shown to have inhibitory activities *in vitro* [25, 26]. However, if there was such activity remaining in our mouse, it would be expected to manifest as a dominant-negative effect and we did not observe any evidence for this.

Mice homozygous for the *Arf4*^*flox*^ allele were viable and showed no detectable phenotypes. To understand the role of Arf4 in the postnatal mouse, we used the tamoxifen-inducible CagCreER driver to delete *Arf4*. This Cre is broadly expressed but remains inactive until mice are treated with tamoxifen [27]. Postnatal day 2 (P2) mice were treated with tamoxifen and genotyped on P10. The viability of animals bearing the *Arf4*^*flox*^*/CagCreER* genotype at P10 was consistently lower than expected from Mendelian distribution, indicating an important role for Arf4 in the early postnatal period. The survivors were notably smaller than littermates (Fig 1B) and had a propensity to die at random times. Necropsy was unremarkable in most cases except for a notable reduction in the size of the pancreas and yellowish feces in the lower intestine. The hair on animals that survived past 6 weeks turned from black to grey (Fig 1C). Since the first anagen phase of the hair cycle occurs at about P28 [28] the hair coming in at this time would have been formed after tamoxifen treatment, thus indicating a role for Arf4 in hair pigmentation. This phenotype is in line with the report by [18] that Arf4 plays an important role in skin cells.

The major cellular phenotype observed in the germline Arf4 knockout mouse was a malformation of the microvilli on the visceral endoderm. Instead of being straight and having a uniform diameter, the mutant microvilli were curved and bulbous [24]. To determine if Arf4 is generally involved in microvilli formation or maintenance, we examined kidney proximal tubules and intestinal epithelia by transmission electron microscopy as these cell types have extensive brush borders. In both organs, the microvilli of the experimental animals appeared normal and had none of the structural defects observed in the visceral endoderm of the germline knockout (S1 Fig).

**Fig 2:**
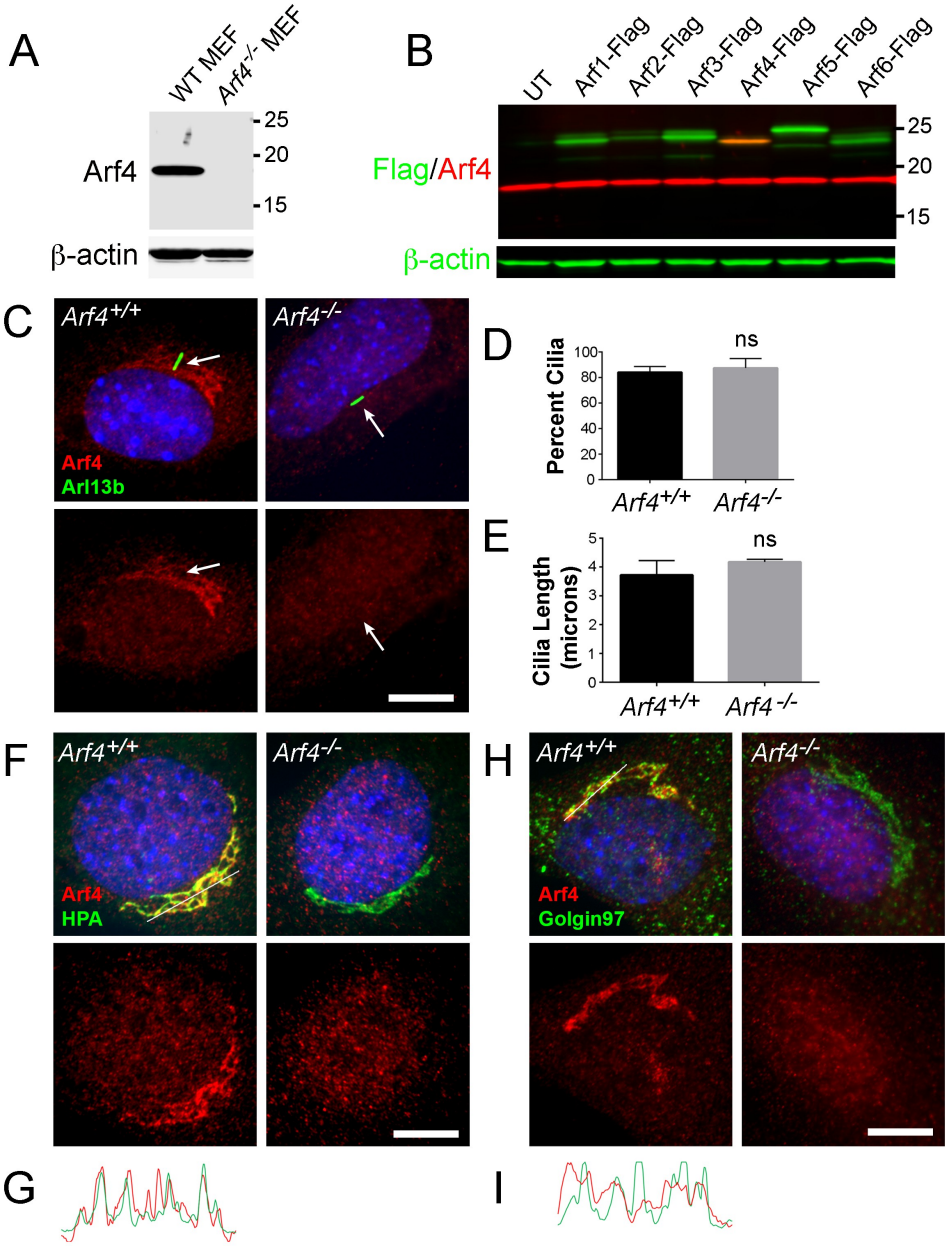
Subcellular localization of Arf4 in mouse embryonic fibroblasts. A. Affinity purified rabbit polyclonal raised against mouse Arf4 detected native Arf4 at ~17kD in wild type MEFs, but not in the *Arf4*^*-/-*^ MEFs. B. Affinity purified rabbit polyclonal raised against mouse Arf4 (red) detected overexpressed Flag-tagged Arf4 but did not react with the other five Flag-tagged mouse Arfs expressed in mIMCD3 cells. Anti-Flag (green) was used to detect the overexpressed Arf-Flags. The anti-Arf4 antibody also detected native Arf4 at ~17kD in the mIMCD3 cells. UT, untransfected mIMCD3. C. Immunofluorescence of wild type or *Arf4*^*-/-*^ MEFs stained with anti-Arf4 antibody (red), cilia marker (anti-Arl13b antibody, green) and nuclei (DAPI, blue). Bottom image of each pair is the Arf4 (red) channel alone. The Golgi-localized Arf4 staining observed in wild type cells is absent in the mutants. Cilia are present in both genotypes and there is no detectable Arf4 in the cilia. Scale bar = 10 μm. D. Graph showing the percent of cells that contained cilia in wild-type and *Arf4*^*-/-*^ cells. N is >100 cells from three MEF lines for each genotype. E. Graph showing length of cilia in wild-type and *Arf4*^*-/-*^ MEFs. Cilia length was measured on 34 cells from three MEF lines per genotype. F. Immunofluorescence of wild type or *Arf4*^*-/-*^ MEFs stained with an anti-Arf4 antibody (red), a cis-medial Golgi compartment marker *Helix pomatia* agglutinin (HPA, green) and nuclei (DAPI, blue). Bottom image of each pair is the Arf4 (red) channel alone. Scale bar = 10 μm. G. Intensity profile of the Arf4 (red) and HPA (green) channels along the line in the top left panel of F. Note the extensive coordination between the peaks in the two channels along most of the line. H. Immunofluorescence of wild type or *Arf4*^*-/-*^ MEFs stained with anti-Arf4 antibody (red), a trans-Golgi compartment marker (anti-Golgin97 antibody, green) and nuclei (DAPI, blue). Bottom image of each pair is the Arf4 (red) channel alone. Scale bar = 10 μm. I. Intensity profile of the Arf4 (red) and Golgin97 (green) channels along the line in H (top left panel). Note the lack of coincidence between the two channels.

To determine the penetrance of the conditional Arf4 knockout in specific tissues and to address the cellular localization of Arf4 in wild type animals, we generated an antibody against a peptide corresponding to the residues 98–114 of mouse Arf4. This sequence has the lowest homology to other Arf family members. This antibody recognized a 17 kDa band corresponding to the endogenous Arf4 in wild type mouse embryonic fibroblasts (MEFs), which was absent in *Arf4*^*-/-*^ MEFs (Fig 2A). To further establish antibody specificity across the members of Arf protein family, we transiently transfected mIMCD3 cells with all six Arf proteins bearing a Flag-tag and found that only endogenous Arf4 and its Flag-tagged recombinant protein were recognized in Western blots of lysed cells (Fig 2B).

Immunofluorescence analysis of wild type MEFs indicated that Arf4 localizes to the Golgi complex and has no association with the cilium (Fig 2C). *Arf4*^*-/-*^ fibroblasts displayed a normal fraction of ciliated cells and normal ciliary length, indicating that Arf4 is not critical to ciliogenesis (Fig 2D and 2E). To identify which compartment of the Golgi was labeled by Arf4, cells were co-stained for Arf4 and either the cis-medial Golgi marker HPA or the trans-Golgi marker Golgin97. Overlap between the cis-medial marker and Arf4 was extensive, while the trans-Golgi marker labeled structures next to the Arf4 staining with minimal overlap (Fig 2F–2I). The Arf4 antibody also produced punctate labeling in the cytoplasm, but identical staining was observed in *Arf4*^*-/-*^ cells suggesting that it represents nonspecific staining (Fig 2C, 2F and 2H). These results indicate that the primary localization of Arf4 in ciliated cells is in the cis-medial Golgi rather than trans Golgi or cilium, a conclusion consistent with localization of GFP-tagged Arf4 [29].

### The loss of Arf4 in the retina does not cause rhodopsin mislocalization or photoreceptor degeneration

**Fig 3:**
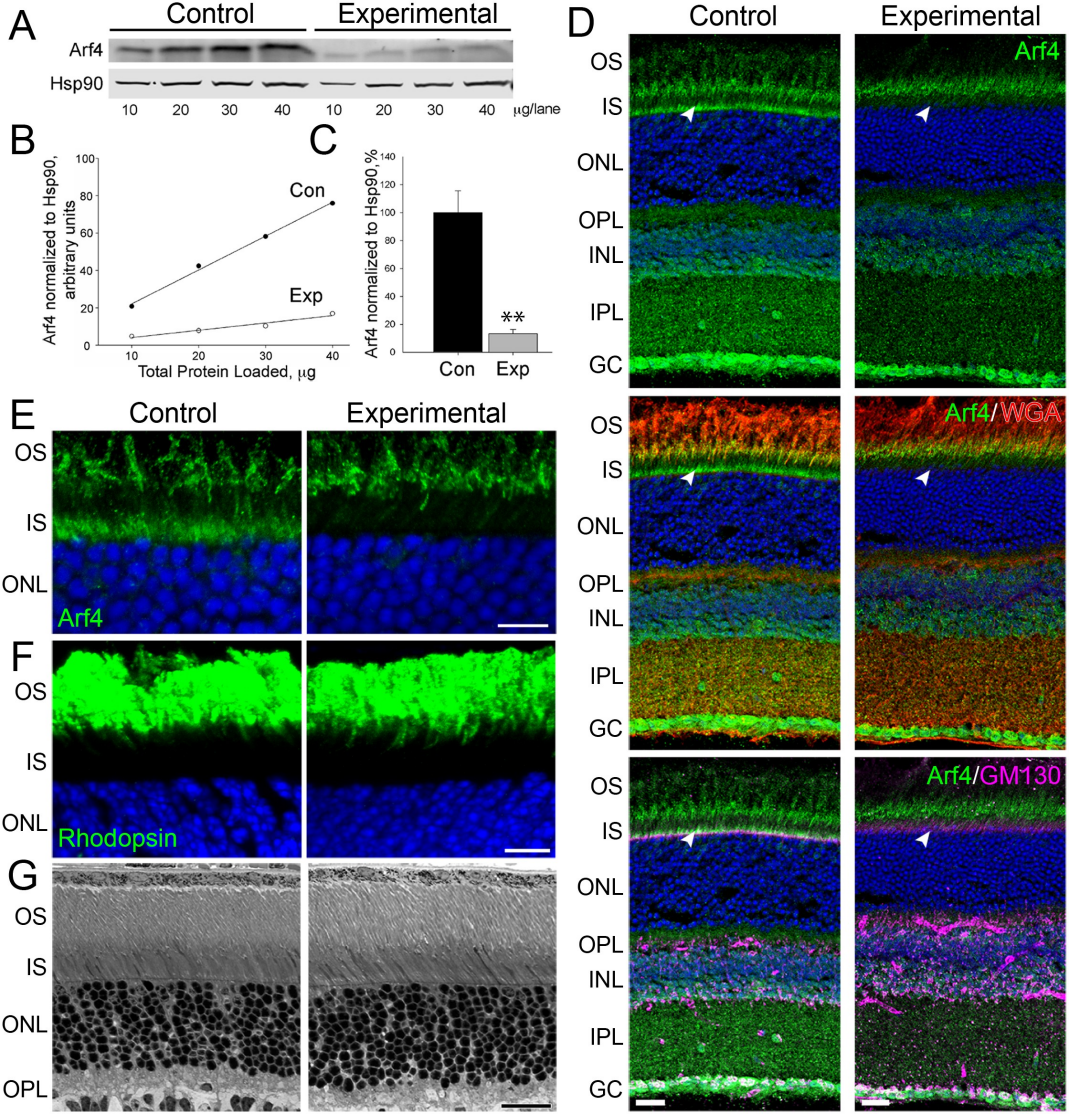
Deletion of Arf4 from the retina does not disrupt rhodopsin localization or photoreceptor morphology. A. Representative Western blots show serial dilutions of control (*Arf4*^*+/flox*^*/CagCreER*) and experimental (*Arf4*^*flox/flox*^*/CagCreER*) eyecup lysates for Arf4 and Hsp90 proteins. Mice were analyzed at P48. B. The fluorescent signal produced by the Arf4 band was normalized to Hsp90 and plotted against total protein loaded. The slope of the curves was used to calculate the amount of each protein in control and experimental eyecups. C. Quantification of Arf4 levels in tamoxifen-treated *Arf4*^*flox*^*/CagCreER* eyecups. Level remaining is relative to Hsp90. **p = 0.001. N = 5 for each genotype, from eyecups collects between P30-P50. D. Immunofluorescence of *Arf4*^*flox*^*/CagCreER* experimental and control retinal cross-sections using anti-Arf4 antibody (green). Specific retinal layers are shown by staining with an extracellular lectin, wheat-germ agglutinin (red, middle panel). Retinal biosynthetic membranes were stained with the Golgi marker anti-GM130 antibody (magenta, bottom panel). The position of Golgi in photoreceptors is marked by arrowhead. In all panels, nuclei stained with Hoechst (blue). Eyes were collected at P34. Scale bar = 20 μm. E. Arf4 immunostaining in *Arf4*^*flox*^*/CagCreER* experimental and control retinal cross-sections. Image of the photoreceptor IS where the biosynthetic membranes are localized. Eyes were collected at P34. Scale bar = 10 μm. F. Rhodopsin immunostaining in *Arf4*^*flox*^*/CagCreER* experimental and control retinal cross-sections. Eyes were collected at P34. Scale bar = 10 μm. G. Comparative analysis of photoreceptor morphology in *Arf4*^*flox*^*/CagCreER* experimental and control retinal cross-sections. Eyes were collected at P41. Scale bar = 20 μm. OS = outer segment, IS = inner segment, ONL = outer nuclear layer, OPL = outer plexiform layer, INL = inner nuclear layer, IPL = inner plexiform layer, GC = ganglion cell layer.

Arf4 involvement in ciliary protein trafficking was first described for the visual pigment, rhodopsin. Therefore, Arf4 knockout was predicted to affect rhodopsin localization and likely photoreceptor health, as rhodopsin mislocalization is associated with retinal degeneration [13, 15]. To test this prediction, we used inducible Arf4 conditional knockout mice (*Arf4*^*flox*^*/CagCreER*) to analyze rhodopsin localization and photoreceptor morphology. We used quantitative Western blotting to measure the relative amounts of Arf4 in the eyecups of experimental and control animals. Serial dilutions of eyecup extracts were analyzed on the same blot (Fig 3A), the densities of Arf4 bands were measured, plotted as a function of total protein amount and fitted with straight lines. The extent of Arf4 reduction in experimental animals was calculated from the ratio between these fits (Fig 3B). This analysis showed that Arf4 in *Arf4*^*flox*^*/CagCreER* eyecups was reduced to ~13% of control (Fig 3C), indicating that the penetrance of tamoxifen induction of the knockout was highly efficient. Considering that activation of Cre recombinase in a given cell results in a complete and irreversible deletion of the *Arf4* gene, this level of protein reduction indicates that Arf4 was completely lost from the majority of cells. Therefore, the small amount of remaining Arf4 originated from cells where Cre failed to delete the gene. Such a mosaic induction pattern is typical for this tamoxifen-inducible model [27].

Arf4 immunostaining of retinal cross-sections from control animals showed a complex staining pattern with strong signals found in all retinal layers (Fig 3D, WGA used to label outer segments and retinal plexiform layers). A side-by-side staining of cross section from *Arf4*^*flox*^*/CagCreER* animals revealed a complete loss of Arf4 from the inner segments of photoreceptor cells, which are localized above photoreceptor nuclei and are partially co-stained with the Golgi marker, GM130 (Fig 3D). On the other hand, the Arf4 staining at the base of the outer segment layer was preserved (Fig 3D and 3E). Considering that outer segments start forming at least one-week post tamoxifen injection and are completely renewed every 12 days, we conclude that Arf4 staining at this location is non-specific. This is consistent with the anti-Arf4 antibody cross-reacting with several additional bands on Western blots obtained from eyecup extracts (S2 Fig). Based on these observations, we conclude that the degree of Arf4 knockout in photoreceptors was essentially complete.

An overall reduction of Arf4 staining was observed in the inner retina as well, although residual signal remained significant at various locations indicating either non-specific staining or resistance of certain cells to tamoxifen treatment (Fig 3D). Regardless, these cells were not the subject of our investigation.

We next investigated whether Arf4 knockout in photoreceptors affects rhodopsin localization by immunostaining retinal cross-sections with an anti-rhodopsin antibody. To our great surprise, rhodopsin was normally localized to rod outer segments of *Arf4*^*flox*^*/CagCreER* animals. No difference was observed between experimental and control animals even when the rhodopsin signal was grossly oversaturated (Fig 3F). Consistent with normal rhodopsin localization, we did not observe any morphological abnormalities in the photoreceptor layer of *Arf4*^*flox*^*/CagCreER* mice, which was further documented using thin plastic retinal cross-sections (Fig 3G).

**Fig 4:**
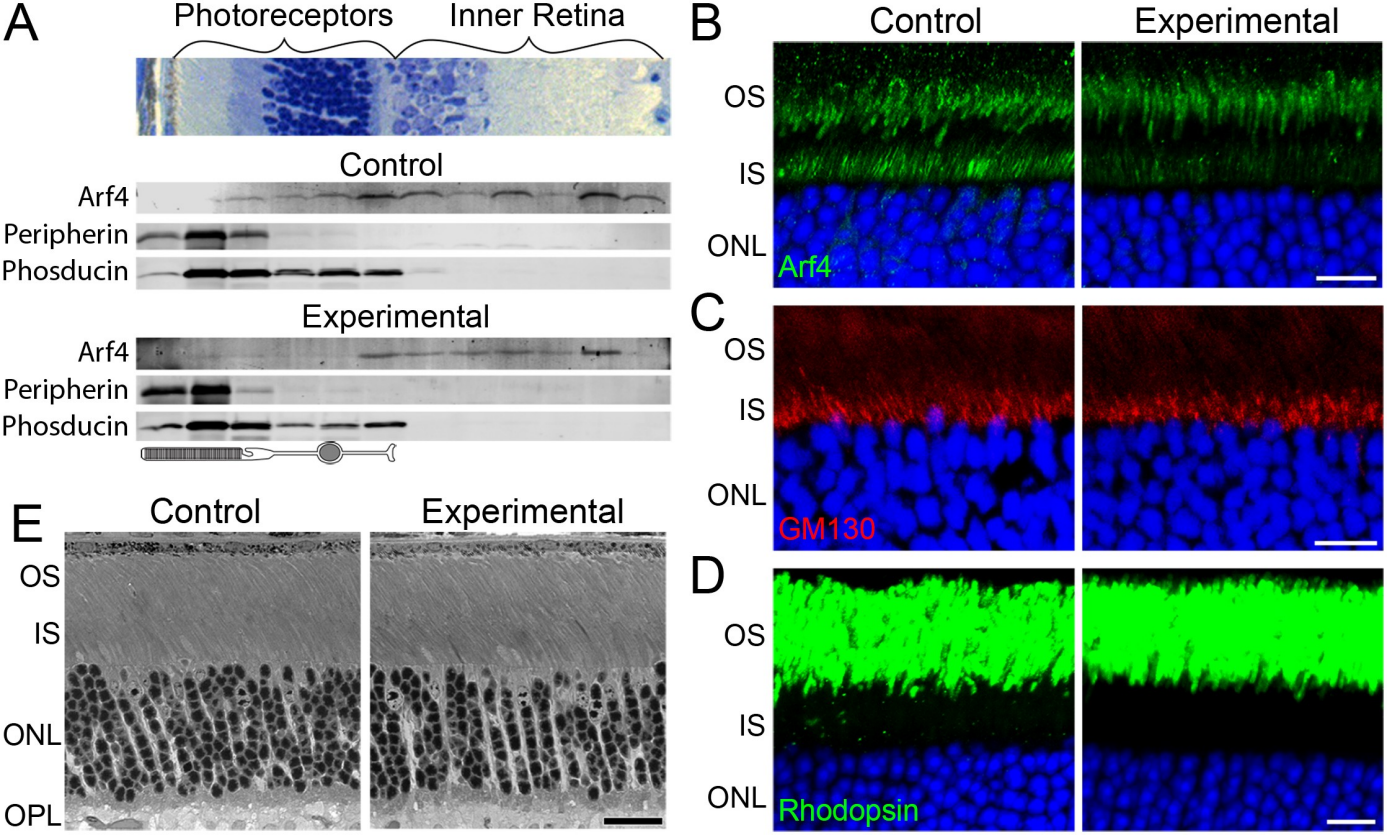
Specific deletion of Arf4 from rod photoreceptors does not affect rhodopsin localization to the ciliary outer segment compartment or photoreceptor morphology. A. The distribution of Arf4 in 20 μm tangential sections through the entire retina of *Arf4*^*+/flox*^*/iCre75* control and *Arf4*^*flox/flox*^*/iCre75* experimental mice. A representative cross-section of a retina is shown above the Western blot panes. Peripherin was used as a marker for the outer segment layer, while phosducin marks the photoreceptor layer. A cartoon of a photoreceptor is used to depict each section. Mice were analyzed at 4 months of age in this and in all other panels. B-D. Immunofluorescence of *Arf4*^*flox*^*/iCre75* experimental and control retinal cross-sections using anti-Arf4 antibody (green, B), the Golgi marker anti-GM130 antibody (red, C) and anti-rhodopsin antibody (green, D). In all panels, nuclei stained with Hoechst (blue). Scale bar = 10 μm. E. Comparative analysis of photoreceptor morphology in *Arf4*^*flox*^*/iCre75* experimental and control retinal cross-sections. Eyes were collected at P60. Scale bar = 20 μm. OS = outer segment, IS = inner segment, ONL = outer nuclear layer.

Given that our result contradicted the central paradigm in rhodopsin trafficking that Arf4 is an indispensable player in this process, we replicated this result by employing an alternative strategy to specifically knock out Arf4 from rod photoreceptor cells. We crossed *Arf4*^*flox*^ mice with iCre75 mice, which express Cre recombinase under control of the rhodopsin promoter [30]. Because Arf4 is expressed in multiple cell types of the retina, Western blot of retinal extracts could not be used to assess the efficiency of Arf4 knockout in rods. Therefore, we resorted to the more sophisticated technique of serial sectioning with Western blotting [31]. We obtained individual 20 μm-thick tangential sections through the entire depth of a flat-mounted frozen retina and determined their Arf4 contents using an anti-Arf4 antibody. Specific sections were assigned to individual photoreceptor layers using two protein markers, peripherin localized in the outer segment [32] and phosducin localized throughout the entire photoreceptor cytoplasm [33]. As shown in Fig 4A, Arf4 was significantly reduced in sections representing the photoreceptor layer, whereas its content in the inner retina was well-preserved. Interestingly, the overall content of Arf4 in the inner retina of control animals was higher than in the photoreceptor layer.

The knockout was further confirmed by a gross reduction of Arf4 immunostaining in photoreceptor inner segments (Fig 4B). One difference from the *Arf4*^*flox*^*/CagCreER* mice was that the Arf4 signal was preserved in a small subset of photoreceptors, which is predicted because a small fraction of photoreceptors are cones not expressing Cre recombinase in this mouse. The outer segment layer staining with anti-Arf4 antibody was also present, reconfirming its non-specificity. Normal staining was observed with the Golgi marker, GM130 (Fig 4C).

The fact that rod-specific Arf4 knockout did not affect mouse health, allowed us to assess rhodopsin localization in older animals than in the *Arf4*^*flox*^*/CagCreER* line. No evidence of rhodopsin mislocalization was observed in mice up to 4 months of age (Fig 4D). Similarly, the conditional Arf4 knockout did not affect the morphology of the photoreceptor layer (Fig 4E). Taken together, the results from the two conditional Arf4 knockout lines provide compelling evidence that Arf4 is entirely dispensable for rhodopsin trafficking and its presence is not necessary for maintaining photoreceptor health.

### The loss of Arf4 in the kidney does not affect ciliogenesis or cause polycystic disease

**Fig 5:**
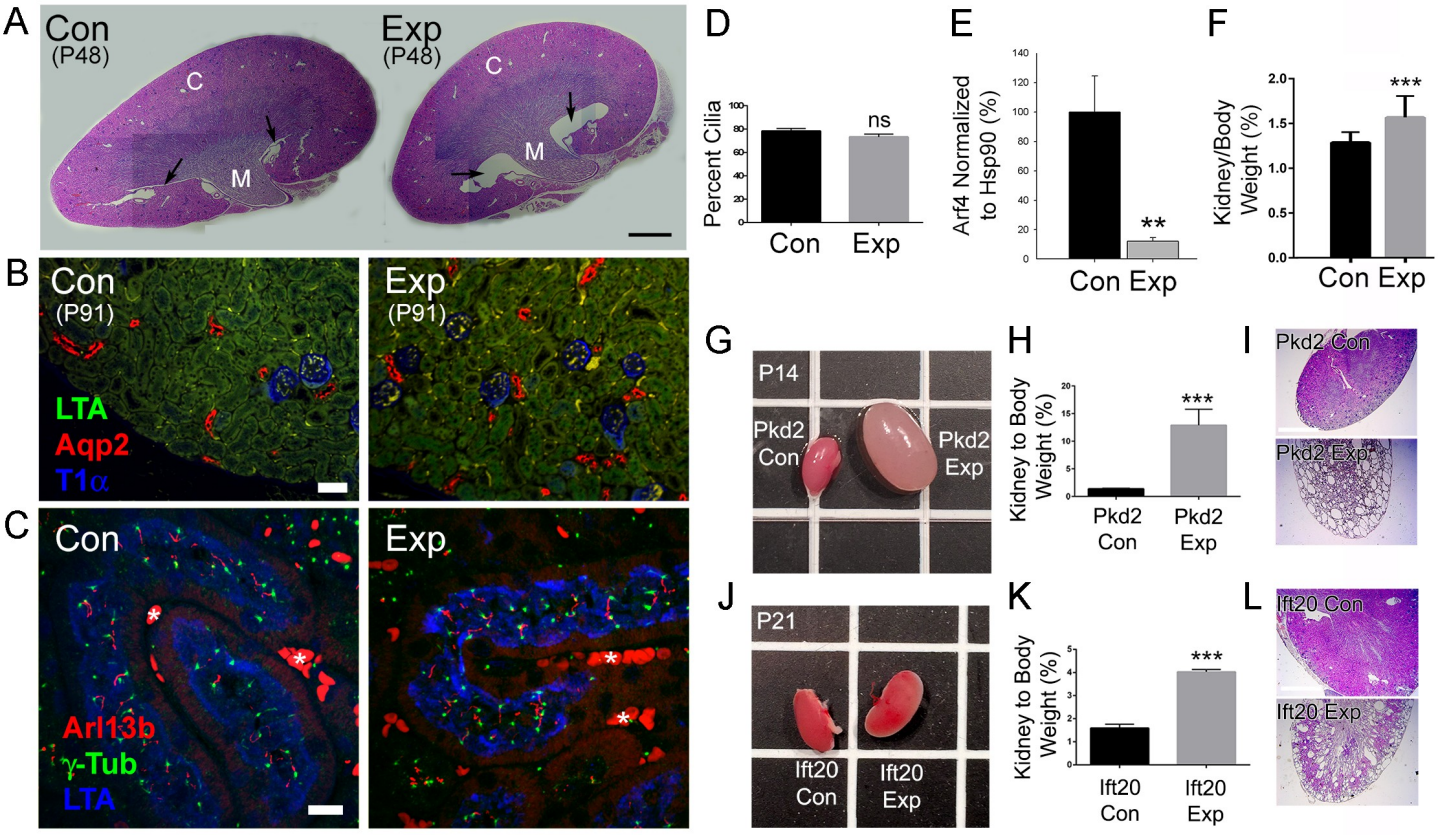
*Arf4* is not a cystic kidney disease gene. A. H&E stained sections of *Arf4*^*flox*^*/CagCreER* kidneys. Note lack of cystic kidney disease, while mild dilation of the renal pelvis or hydronephrosis (arrows) was observed in 7 of 7 experimental animals examined. Kidneys were collected at P48. Each kidney is a composite of 8 images. Scale bar = 1 mm. B. Kidney sections of *Arf4*^*flox*^*/CagCreER* control and experimental animals stained with a proximal tubule marker (LTA, green), collecting duct marker (aquaporin-2, red) and the renal corpuscle marker (T1α, blue). Kidneys were collected at P91. Scale bar = 50 μm. Green is mostly autofluorescence. C. Kidney sections of *Arf4*^*flox*^*/CagCreER* control and experimental mice stained for cilia (Arl13b, red), centrosome (gamma tubulin, green) and proximal tubule (LTA, blue) markers. Asterisks mark red blood cells. Scale bar = 10 μm. D. Percent cilia in proximal tubules of *Arf4*^*flox*^*/CagCreER* animals. Percent cilia was determined by examining Z-stacks of proximal tubules stained with centrosome and cilia markers and presented as percent of centrosomes (or centrosome pairs in G2 cells) that are ciliated. N = >100 centrosomes from three animals of each genotype. ns, not significant. E. Quantification of Arf4 levels in tamoxifen-treated *Arf4*^*flox*^*/CagCreER* kidneys. The contralateral kidney from control and experimental animals used for histological studies was analyzed for Western blotting. Level remaining is relative to Hsp90. **p = 0.011. N = 4 for each genotype. F. Quantification of total kidney weight to body weight. Data was pooled from the animals shown in Fig 1B. ***p<0.0001 with respect to controls. G. Image of *Pkd2*^*flox*^*/CagCreER* control and experimental kidneys at P14. Grid lines are 1.35 cm apart. H. Kidney to body weight comparisons of *Pkd2*^*flox*^*/CagCreER* control and experimental animals at P14. N = 9 experimental and 28 controls. ***p<0.0001 with respect to controls. I. H&E image of *Pkd2*^*flox*^*/CagCreER* control and experimental kidneys at P14. Scale bar is 1 mm. J. Image of *Ift20*^*flox*^*/CagCreER* control and experimental kidneys at P21. Grid lines are 1.35 cm apart. K. Kidney to body weight comparisons of *Ift20*^*flox*^*/CagCreER* control and experimental animals. N = 4 for both genotypes. ***p<0.0001 with respect to controls. L. H&E image of *Ift20*^*flox*^*/CagCreER* control and experimental kidneys at P21. Scale bar is 1 mm.

Ward et al. [17] showed that knockdown of Arf4 in cell culture reduces steady state levels of ciliary polycystin-1, and we found a reduced rate of fibrocystin delivery to cilia when Arf4 was knocked down [24]. These results suggest that the lack of Arf4 should produce a cystic kidney phenotype. To test this, we analyzed kidneys from *Arf4*^*flox*^*/CagCreER* mice injected with tamoxifen at P2 (Fig 5A–5F). In CagCreER mice, Cre is expressed in all segments of the tubule [27] and previous work has shown that deletion of cilia genes prior to ~P14 leads to rapid cyst formation [34, 35]. To ensure that this protocol is capable of driving cyst formation, the same regiment was used with *Pkd2*^*flox*^*/CagCreER* mice and *Ift20*^*flox*^ */CagCreER*. Severe cystic disease developed by P14 in *Pkd2*^*flox*^*/CagCreER* mice and by P21 in *Ift20*^*flox*^ */CagCreER* mice (Fig 5G–5L), indicating that this protocol is sufficient to induce cysts in susceptible mice.

*Arf4*^*flox*^*/CagCreER* treated mice were collected at various ages between P10 and P91 (see Fig 1B). Western blot analysis showed that Arf4 was reduced to about 15% of normal in the experimental kidneys (Fig 5E and S3 Fig), indicating that the majority of kidney cells lacked *Arf4*. Kidney to body weight was slightly larger in the experimental animals (Fig 5F) but no evidence of cystic enlargement in any portion of the tubule was observed (Fig 5A and 5B). A small expansion of the renal pelvis, or hydronephrosis, was observed in the experimental animals (Fig 5A). This may be enough to account for the slightly larger kidney to body weight. However, the experimental animals were undersized, which could also account for this difference if not all organs were equally growth-retarded. No defects were observed in the cilia of experimental mice (Fig 5C) and the fractions of ciliated proximal tubule cells were also equal in both groups (Fig 5D).

**Fig 6:**
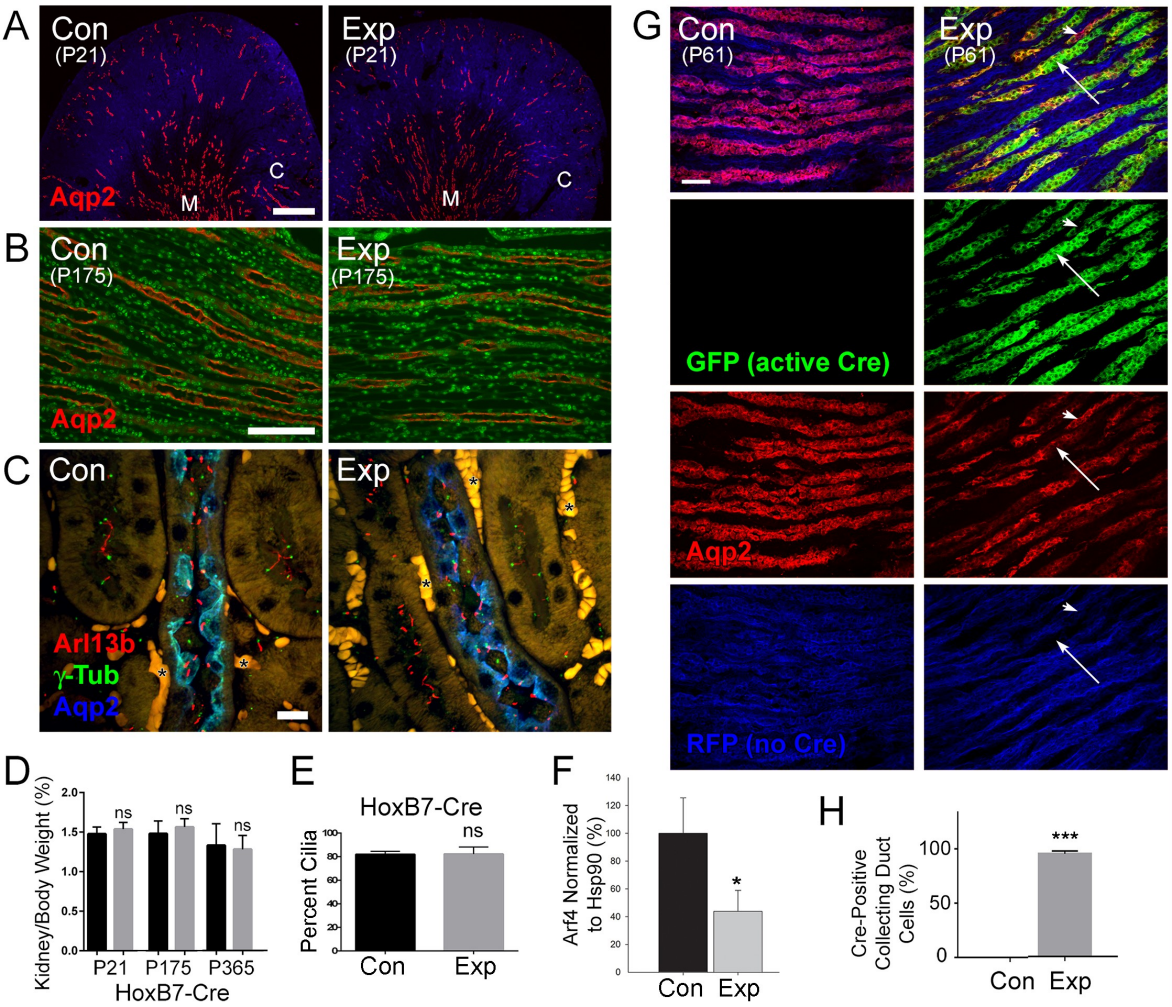
Specific deletion of *Arf4* from collecting ducts with HoxB7-Cre does not cause cystic kidney disease gene. A-B. Kidney sections of *Arf4*^*flox*^*/HoxB7-Cre* control and experimental animals stained for collecting duct marker (aquaporin-2, red) and DNA (DAPI, blue in A, green in B). Kidneys in A were collected at P21 and in B were collected at P175. M = medulla, C = cortex. Scale bar in A = 500 μm, in B = 100 μm. C. Kidney sections of *Arf4*^*flox*^*/HoxB7-Cre* control and experimental mice stained for cilia (Arl13b, red), centrosome (gamma tubulin, green) and collecting duct (aquaporin-2, blue) markers. * marks red blood cells. Scale bar = 10 μm. Each image is a maximum projection of 16 confocal images taken at 0.5 μm intervals. D. Quantification of total kidney weight to body weight. *Arf4*^*flox*^*/HoxB7-Cre* animals are separated by age group. N = 5–7 animals for each genotype and age. ns, not significant with respect to controls at same age. E. Percent cilia in collecting ducts of *Arf4*^*flox*^*/HoxB7-Cre* animals. Percent cilia was determined by examining Z-stacks of proximal tubules stained with centrosome and cilia markers and presented as percent of centrosome (or centrosome pairs in G2 cells) that are ciliated. N = >100 centrosomes from three animals of each genotype. Difference is not significant. F. Quantification of Arf4 levels in *Arf4*^*flox*^*/HoxB7-Cre* kidneys. The kidney papilla, which is about 50% collecting duct cells, was dissected from the rest of the kidney and analyzed by Western blotting. Level remaining is relative to Hsp90. *p = 0.01. N = 4 for each genotype. G. The mTmG Cre reporter was crossed into the *Arf4*^*flox*^, *HoxB7-Cre* line. With this reporter, Cre activity switches expression of RFP (blue) to GFP (green). HoxB7-Cre is active in collecting ducts, which were marked by aquaporin-2 (red). Note the absence of GFP-positive collecting duct cells from Cre negative animals whereas most collecting duct cells are GFP positive in experimental animals expressing HoxB7-Cre. Large arrow marks GFP-positive cells while the arrow head marks a cell that was not converted from RFP to GFP. Scale bar = 50 μm. Each image is a maximum projection of 11 confocal images taken at 1 μm intervals. H. Quantification of Cre-active collecting ducts cells. ***p<0.0001, n = 3 animals of each genotype >400 cells counted per animal.

Since the failure to observe cyst formation in our *Arf4*^*flox*^*/CagCreER* animals was unexpected, we used HoxB7-Cre as a second method to delete *Arf4* (Fig 6). HoxB7-Cre is active in kidney collecting ducts [36]. Previous work showed that HoxB7-Cre-driven deletion of cilia genes results in severe cystic kidney disease with kidneys becoming ~10 fold larger than normal by P21 [37, 38]. Kidneys from *Arf4*^*flox*^*/HoxB7-Cre* experimental and control mice were collected at P21, P175 and P365 (Fig 6A–6E). Western blot analysis showed that Arf4 in experimental papilla was reduced to about 40% of normal (Fig 6F and S3 Fig). Because the papilla is composed of about 50% collecting duct cells (where Arf4 was deleted), with the rest being represented by cells from the thin loops of Henley, the interstitium and the vasa recta capillary bed, we conclude that the knockout penetrance was extremely deep. No evidence of cystic expansion of the collecting ducts was observed by histology in any experimental animals (Fig 6A and 6B). Furthermore, the ratio of kidney to body weight was not significantly different between the control and experimental mice at any age (Fig 6D). Normally, the kidney weighs about 1.5% of the total body weight, but the deletion of *Ift20* by the same Cre driver increases this number to 16% at P21 [37]. In addition, no defects in cilia were observed (Fig 6C) and equal percentages of collecting duct cells were ciliated in both groups (Fig 6E). To ensure that the HoxB7-Cre driver used for these experiments was active, we crossed the mTmG Cre reporter [39] into the line. This reporter expresses red fluorescent protein that is converted to green fluorescent protein upon Cre recombination. Control animals lacking Cre contained no GFP-positive collecting ducts while the vast majority of collecting duct cells were GFP positive in the experimental animals, which expressed HoxB7-Cre (Fig 6G and 6H). Overall, our data indicate that Arf4 is dispensable for maintaining normal kidney structure and its loss does not lead to cystic kidney disease.

### Loss of Arf4 causes severe degeneration of the exocrine pancreas

**Fig 7:**
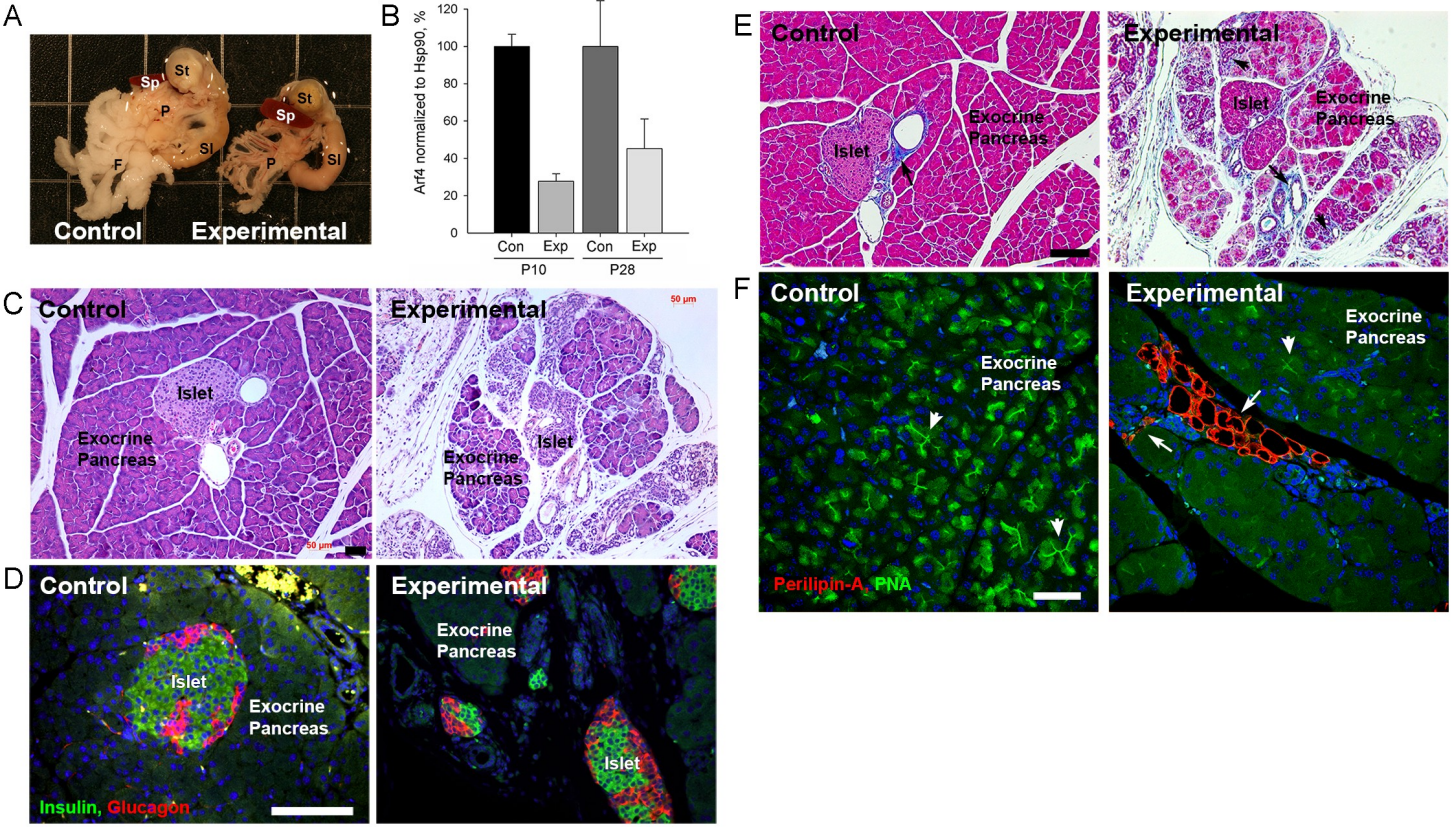
Postnatal deletion of Arf4 causes exocrine pancreas degeneration. A. Pancreas (P) dissected from a P91 control and experimental *Arf4*^*flox*^*/CagCreER* siblings. Note the smaller pancreas in the experimental compared to the control and the lack of the mesenteric fat pad (F) that normally associates with the pancreas. St = stomach, SI = small intestine. Grid lines are 1.35 cm apart. B. Quantification of Arf4 levels in *Arf4*^*flox*^*/CagCreER* pancreas at two developmental time points, P10 and P28. Arf4 remaining was estimated to be 27% of normal at P10 and 45% at P28. Level remaining is relative to Hsp90. N = 4 for each genotype. C. H&E stained sections of control and experimental *Arf4*^*flox*^*/CagCreER* pancreas. Note the endocrine pancreas (Islet) is normally embedded in the exocrine pancreas but in the experimental animal the islets are separated from the exocrine cells and surrounded by fat and connective tissue. Scale bar = 50 μm. D. Sections of control and experimental pancreas *Arf4*^*flox*^*/CagCreER* stained for beta cells (insulin, green), alpha cells (glucagon, red) and DNA (DAPI, blue). As with the H&E images, note that the islets have lost their tight association with the exocrine pancreas. Scale bar = 100 μm. E. Trichrome blue stained sections of control and experimental *Arf4*^*flox*^*/CagCreER* pancreas. Note in control and experimental pancreas, trichrome blue-positive stain is observed near the islet-associated blood vessels (arrow) but in the experimental pancreas positive stain is distributed through the exocrine pancreas (arrowhead) indicating extensive fibrosis. Scale bar = 100 μm. F. Sections of control and experimental pancreas *Arf4*^*flox*^*/CagCreER* stained for peanut agglutinin (PNA, green), lipid droplets (perilipin-A, red) and DNA (DAPI, blue). Note the clusters of perilipin-A-positive adipocytes (arrows) in the experimental animals and the lack of these cells in the control. PNA labels zymogen granules in acinar cells and the intercalated ducts connecting the acinar cells. Arrowheads mark examples where the zymogen granules and the duct are visible. Note the loss of PNA label in the experimental pancreas as compared to the control. Scale bar = 50 μm. Image is a maximum projection of a 10 image z-stack taken at 0.5 μm intervals.

During necropsy of *Arf4*^*flox*^*/CagCreER* animals we noted that their pancreas was abnormally small (Fig 7A). In addition, experimental pancreases were opaque and had islet-sized spheres visible within the tissue, suggesting exocrine pancreas abnormality. The amount of Arf4 remaining in these pancreases was estimated to be 27% of normal at P10 and 45% at P28 (Fig 7B and S4 Fig). The apparently smaller Arf4 reduction than in the kidney and Arf4’s increase with age can be explained by ongoing degeneration of cells in which Cre recombinase was induced. Accordingly, the fraction of non-induced cells in these pancreases increases with age. Histological examination revealed that islets in experimental pancreas appear normal but the surrounding exocrine tissue is reduced in volume, fragmented and partially replaced by what appears to be fibrotic material and adipocytes (Fig 7C). Immunofluorescence with endocrine markers confirmed that the experimental islets were comparable to the control islets and both had similar distributions of alpha and beta cells (Fig 7D). Trichrome blue, which labels collagen, normally highlights the tunicae surrounding the vasculature of the pancreas. This staining was observed in both experimental and control animals, but the experimental animals also showed extensive labeling within the field of secretory acini suggestive of developing fibrosis (Fig 7E).

H&E staining suggested that adipocytes may be interspersed within the experimental pancreas (Fig 7C). The exocrine pancreas is associated with a fat pad but the adipocytes are normally only found around the edges of the pancreas and not infiltrated within the field of acini. Accordingly, perilipin-A (a protein located on the surface of lipid droplets within adipocytes) is essentially absent from the control exocrine pancreas. In contrast, perilipin-A-positive cells are found throughout the field of acini in the *Arf4* deleted pancreas (Fig 7F). Fatty pancreas or pancreatic steatosis can be caused by infiltration of adipocytes or transdifferentiation of exocrine cells into adipocytes [40, 41]. Infiltration appears to be driving the fat formation within the pancreas when Arf4 is absent as we did not observe cells that were positive for both perilipin-A and exocrine markers. However, the degenerating *Arf4*-defective cells lose exocrine markers early in degeneration and so it is possible they are transdifferentiating but do not simultaneously express both zymogen granule markers and lipid droplet markers.

**Fig 8:**
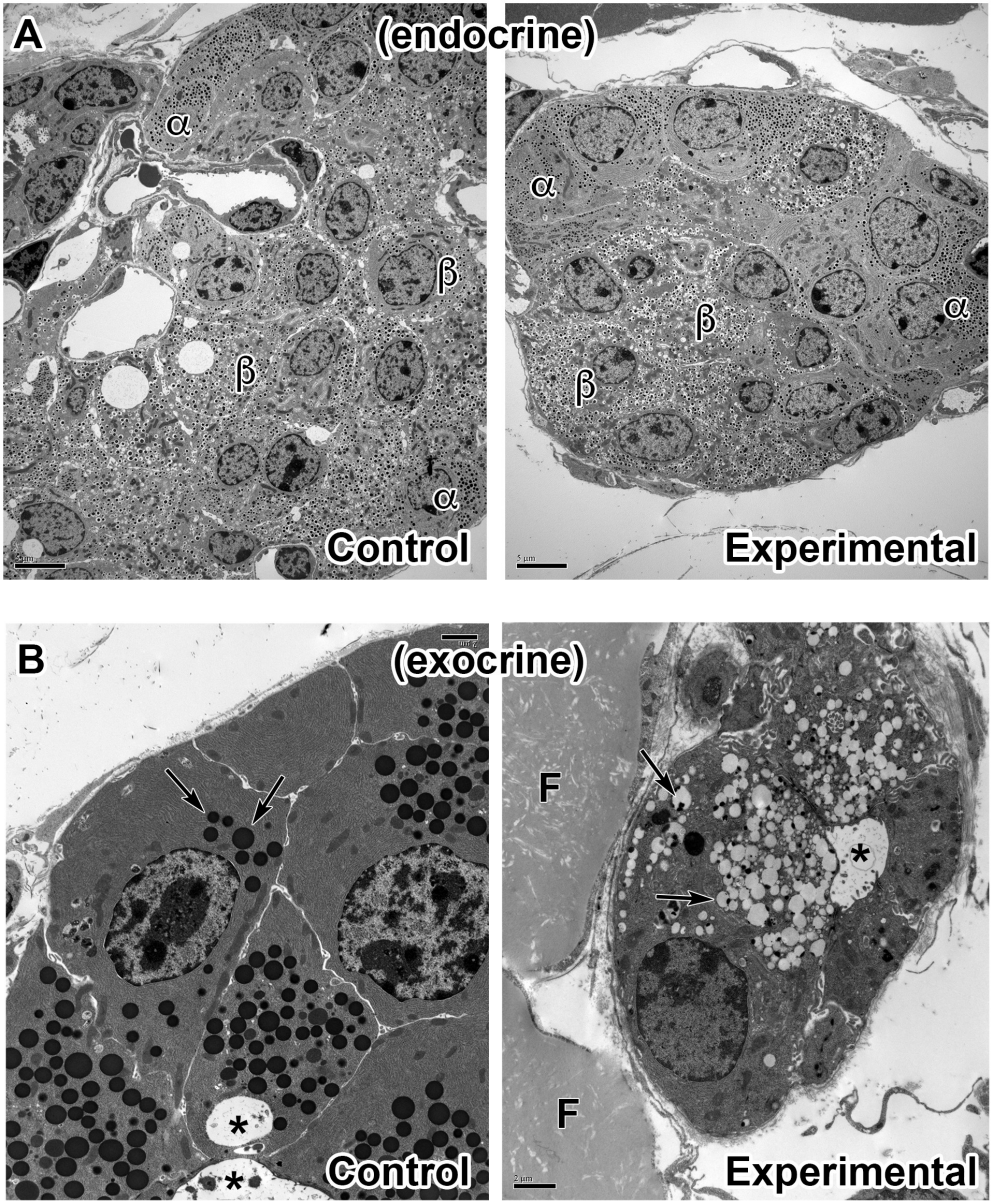
Electron microscopy of the pancreas. A. Transmission electron micrographs of islets from control and experimental *Arf4*^*flox*^*/CagCreER* pancreas. Insulin-producing beta cells (β) are marked by insulin containing granules with clear halos. Glucagon-producing alpha cells (α) are marked by similar-sized granules lacking the clear halos. No differences were detected in either the beta or alpha cells between the two genotypes. Scale bar = 5 μm. B. Transmission electron micrographs of control and experimental *Arf4*^*flox*^*/CagCreER* exocrine cells. Note the large electron dense zymogen granules (arrows) in the control image. Most experimental exocrine cells showed evidence of degeneration including vacuolization of the zymogen granules (arrows). F marks fat cells, * marks the lumen of acini. Scale bar = 2 μm.

To better understand the pancreatic pathology in Arf4 knockout animals, sections from the *Arf4*^*flox*^*/CagCreER* pancreases were examined by electron microscopy. Consistent with light microscopy observations, the islets of the endocrine pancreas appeared unaffected. The alpha and beta cells looked normal with abundant glucagon and insulin granules inside (Fig 8A). In contrast, the acinar cells of the exocrine pancreas were variably affected in knockout mice and surrounded by adipocytes with large intracellular lipid droplets (Fig 8B). In control animals, acinar cells are densely populated with spherical electron-dense zymogen granules, up to 1 micron in diameter, located near the apical end of the cell surrounding the central duct. In experimental animals, some acinar cells looked normal while others showed vacuolization of the zymogen granules (Fig 8B). Electron dense granules in these cells were smaller than normal and associated with larger translucent 0.25 to 1 micron spheres.

## Discussion

Arf4 first came to interest as a ciliary trafficking regulator when it was found to bind to the C-terminus of rhodopsin, which encodes the outer segment targeting signal. *In vitro* experiments demonstrated that perturbing Arf4 could inhibit the formation of rhodopsin carrier vesicles from Golgi membranes [21]. Subsequent work suggested an interaction network for Arf4, including ASAP1, Rab11 and other proteins [10], that appear to be important for the trafficking of rhodopsin constructs to primary cilia of cultured cells [42]. The only published validation of this model *in vivo* was performed with frog photoreceptors after transgenic expression of Arf4-GFP or its dominant negative Arf4^I46D^-GFP mutant [16]. Photoreceptor cells expressing the mutant had abnormal cell junctions, perturbed Golgi membranes and partially mislocalized rhodopsin. The authors interpreted these observations as evidence for Arf4 involvement in rhodopsin trafficking, but these effects could be a consequence of a generalized Golgi breakdown caused by the overexpression of Arf4^I46D^-GFP mutant. The authors also reported that expression of Arf4^I46D^-GFP causes retinal degeneration. However, in this experiment the appearance of rods expressing the Arf4^I46D^-GFP construct is identical to adjacent rods not expressing the construct (Fig 5E in [16]), with only obvious difference between the retinas of Arf4-GFP and Arf4^I46D^-GFP frogs being the amount of the outer segment covered by the RPE (Fig 5D vs. Fig 5E in [16]).

### Arf4 is dispensable for rhodopsin trafficking in photoreceptors

In this study, we directly addressed whether Arf4 is required for rhodopsin trafficking by knocking it out from photoreceptors using two independent Cre-lox systems. We found that neither mouse line displayed even the slightest abnormality in subcellular rhodopsin localization, demonstrating that Arf4 is entirely dispensable for rhodopsin processing by the biosynthetic membranes, outer segment targeting and delivery. Consistent with normal rhodopsin trafficking, photoreceptors of knockout mice displayed no structural abnormality or signs of degeneration. One explanation for our negative result is that rhodopsin trafficking in photoreceptors follows an Arf4-independent route, such as intraflagellar transport (reviewed in [9]). Of particular interest in this context is the role of Ift20, a dynamic component of the IFT complex B, which localizes at both Golgi and at the ciliary base [43, 44]. This positions Ift20 to recruit and guide rhodopsin transport vesicles from the biosynthetic membranes to the cilium. Accordingly, inducible knockout of Ift20 caused rhodopsin accumulation in the Golgi membranes supporting a role for Ift20 in sorting or transporting rhodopsin from Golgi to the outer segment [45, 46].

### *Arf4* is not a cystic kidney disease gene

Arf4 was predicted to be a cystic kidney disease gene based on polycystin-1 [17] and fibrocystin [24] trafficking defects *in vitro*. Ward and colleagues showed that Arf4 bound to a VxPx motif in the C-terminal tail of polycystin-1 that was required for targeting of the protein to cilia. They further showed that knockdown of Arf4 in cell culture prevented polycystin-1 from localizing to cilia. We identified a short motif in fibrocystin that can direct GFP to cilia and found that this peptide bound strongly to Arf4 even though it did not contain a VxPx motif. Knockdown of Arf4 delayed the delivery of a fibrocystin-GFP fusion protein to cilia but did not affect its steady state level [24, 47]. In our current work we directly tested the role of Arf4 in cystogenesis by deleting the gene from the kidney using two different strategies. While these approaches yield cystic kidneys when other cilia genes are deleted neither yielded any evidence for cystogenesis when Arf4 was deleted. This indicates that if Arf4 is important in the kidney, other proteins can compensate in its absence.

### Presence of a VxPx motif is not a predictor of ciliary targeting

The VxPx motif, first identified in rhodopsin [48], has been proposed as a predictor of ciliary localization [2] and VxPx motifs have been found in several ciliary proteins. The best studied example besides rhodopsin, is polycystin-2, which is directed to cilia via the R_6_VxP motif in the N-terminal part of the protein [19]. A VxPx is also important in the outer segment targeting of RDH8, a lipidated retinol dehydrogenase located in outer segment disc membranes [49], as well as the delivery of CNGB1b to olfactory cilia [20]. Arf4 was reported to bind to a VxPx motif in the C-terminal tail of polycystin-1 that was thought to be the ciliary targeting sequence for this protein [17]. Subsequent work failed to reproduce the requirement for the VxPx motif in targeting polycystin-1 to cilia and found no effects on ciliary polycystin-1 when Arf4 was depleted [50]. The motif is also found in CRMP-2 and Nphp3 but mutational analysis indicates that the motif is not required for ciliary localization [51, 52]. To further assess the value of the VxPx motif to predict ciliary localization, we analyzed the prevalence of this motif in the mouse proteome. Predicted frequency is 1 motif every 292 amino acids based on 6.6% V and 5.2% P in the average protein [53]. Similar to predictions, on average we found 1 motif every 237 amino acids and 58% of mouse proteins contained the motif. Since the best guesses of the ciliary complexity suggests that 5% or less of the proteins in the mouse are associated with cilia, finding a VxPx motif in a protein has no predictive value in terms of cellular localization. This is further compounded by the observations that ciliary targeting sequences in fibrocystin and multiple GPCRs do not contain VxPx motifs.

### Arf4 plays a critical role in maintaining the exocrine pancreas

While our work clearly demonstrated the absence of Arf4 does not cause retinal degeneration or cystic kidney disease, Arf4 is a critical protein in post-natal mouse development. In addition to augmented hair color, growth restriction and early death, the most striking phenotype observed in our study is severe degeneration of the exocrine pancreas. The acinar cells of the exocrine pancreas degenerated leaving the islets surrounded by adipocytes and fibrotic material rather than being embedded in exocrine tissue. Fatty pancreas, which goes by the names of pancreatic steatosis or pancreatic lipomatosis, is a significant human pathology [54]. In obesity-associated disease, the adipocytes are believed to infiltrate the pancreas [40], although transdifferentiation of the exocrine cells into adipocytes is also possible upon the loss of cMyc [54]. In the cMyc-driven transdifferentiation, both lipid droplets and zymogen granules are observed in the same cells. In our case, we do not see lipid droplets and zymogen granules together in a cell. However, the exocrine cells appear to lose markers of zymogen granules early in the degeneration process, so it is possible that transdifferentiation is occurring.

Mutations causing exocrine degeneration and fibrosis have been described in a number of mouse models, including other components of the secretory system such as Sec23. *Sec23* encodes a subunit of the COPII complex involved in transport between ER and Golgi. Similar to our observations in the Arf4 knockout, *Sec23*-defective mice have reduced zymogen granules [55]. However, these mice also showed very distended ER, which was not observed in our *Arf4* knockout animals. Exocrine pancreas degeneration has also been described in several mouse models with ciliary defects including *Ift88* and *Pkd1* [56–58]. These mice share the phenotypes with *Arf4* in that the exocrine pancreas is primarily affected without structural defects in the endocrine pancreas. However, the degeneration in the ciliary-related mutations starts with cyst formation in the exocrine ducts, which are not observed in the *Arf4*-defective pancreas.

In summary, we have demonstrated that the loss of Arf4 from the mouse kidney and retina does not recapitulate phenotypes that would be expected if Arf4 was critical for sorting or transporting proteins to the cilium and the outer segment. However, the embryonic lethality that we observed with a germline mutation and the post-natal lethality observed in conditional allele supports critical role for Arf4 in transport through the endomembrane system in specific organs, most strikingly the exocrine pancreas.

## Materials and methods

### Mouse

Arf4^flox^ mice were generated at the Duke Transgenic Mouse Facility. LoxP sites were inserted upstream and downstream of *Arf4* exon 2 and 3 using standard BAC Recombineering techniques. Neo selection was used to select positive embryonic stem (ES) cell clones that were then validated by both Long Range PCR and Southern blot. Validated ES clones were then injected into the 8-cell embryo stage, using the VelociMouse method as described in [59], to generate Arf4^flox^ chimeras. Chimeras were breed to C57Bl6/J mice to produce Arf4^+/flox^ mice. Mice were maintained as C57Bl6/J congenics. Genotyping was done with cArf4-KO-F gggaggattgggaagacaat, cArf4-KO-R1 caccacttgactgggaaggt and cArf4-KO-F2 agcagcctcattgtcctagc, which produces a 400 bp product in wild type and a 268 bp product in the Arf4^flox^ allele. HoxB7-Cre [36] and CagCreER [27] were obtained from Jackson Laboratory. iCre75 [30] were a generous gift from Neena Haider, Schepens Eye Research Institute.

For post-natal deletion of Arf4^flox^ by CagCreER, 5 microliters of 20 mg/ml (0.1 mg) tamoxifen dissolved in corn oil was administered by intraperitoneal injection on P2.

All mouse work was approved by institutional animal use committees at Duke University (protocol A254-16-12) or University of Massachusetts Medical School (protocol A-1174) as regulated by the National Institutes of Health Office of Laboratory Animal Welfare.

### VxPx motif identification

EMBOSS patmatdb was run against the ENSEMBL GRCm38 protein collection. Of the 56999 proteins in this proteome, 33260 (58%) had at least one VxPx motif and the average protein had 2.51 motifs. The average occurrence was 1 motif every 237 amino acids. Predicted frequency is 1 motif every 292 amino acids (based on 6.6% V and 5.2% P in the average protein [53]).

### Cell culture

MEFs were generated from E14 embryos from pregnant mouse treated with 100 microliters of 10 mg/ml (1 mg) tamoxifen (by oral gavage) 48 hr before harvest. Cells were immortalized with Large T antigen and cloned to generate control (*Arf4*^*flox/flox*^*/CagCreER*) and experimental (*Arf4*^*-/-*^*/CagCreER*) lines. MEFs were cultured in 90% DMEM (4.5 g/L glucose), 10% fetal bovine serum, 100 U/ml penicillin, and 100 μg/ml streptomycin (all from Gibco-Invitrogen, Grand Island, NY). SV40 Large T immortalized cells were used for analysis.

mIMCD3 [60] cells were cultured in DMEM/F12 with 10% fetal bovine serum, 100 U/ml penicillin, and 100 μg/ml streptomycin and transfected with Flag-tagged Arf constructs as described in [24].

Cells for immunofluorescence staining were grown on glass coverslips. Serum was reduced to 0.25% for 2 days before fixation to enhance ciliation [43, 61].

### Histology

#### Hematoxylin and eosin

Paraffin sections for H&E staining were dewaxed with Safeclear (Fisher Scientific, Hampton, NH) and rehydrated with graded aqueous solutions of isopropanol. The sections were stained for 4 min with CAT Hematoxylin (Biocare Medical, Concord, CA), rinsed in running tap water for 30 sec followed by three quick dips in saturated lithium carbonate and a rinse in distilled water. This was followed by 90% ethanol for 2 min, Edgar Degas Eosin (Biocare Medical) for 2 min and 3 quick rinses in 100% ethanol. The sections were cleared with Safeclear (two 5 min incubations) and were mounted with Permount (Fisher Scientific).

#### Trichrome blue

Paraffin sections were stained by the Masson’s Trichrome method following manufacturer’s procedure (Poly Scientific R&D Corp, Bay Shore, NY).

#### Toluidine blue

Mouse eyes were fixed in 2% paraformaldehyde / 2% gluteraldehyde in 0.1M Cacadylate Buffer for 2 hr, at 22**°**C. Plastic-embedded retinal cross-sections (1 μm) were prepared as in [33] and stained with toluidine blue for light microscopy.

#### Electron microscopy

Animals were deeply anesthetized and perfused with PBS until the blood was removed followed by 15 min perfusion with 2.5% glutaraldehyde, 2% paraformaldehyde in 100 mM cacodylate pH 7.2. Tissue was then removed and cut in 1 mm cubes or rings and fixed overnight in the same solution before osmication with 1% osmium tetroxide for 1 hr, at 22**°**C. Samples were then rinsed and then dehydrated through a graded ethanol series of 20% increments, before two changes in 100% ethanol. Samples were then infiltrated first with two changes of 100% Propylene Oxide and then with a 50%/50% propylene oxide / SPI-Pon 812 resin mixture. The following day three changes of fresh 100% SPI-Pon 812 resin were done before the samples were polymerized at 68^°^C in plastic capsules. The samples were then sectioned (1 μm) and stained with toluidine blue to locate the islets. The samples were then re-trimmed with the islets in the center, and thin sections of approximately 70 nm were placed on copper support grids and contrasted with Lead citrate and Uranyl acetate. Sections were examined using the FEI Tecani 12 BT with 80Kv accelerating voltage, and images were captured using a Gatan TEM CCD camera.

#### Immunofluorescence

Paraffin sections were dewaxed, rehydrated and subjected to antigen retrieval in an autoclave (250°F, 40 min) with 10 mM sodium citrate at pH 6. Frozen sections of kidneys for native fluorescence were prepared by flash freezing unfixed tissue embedded in Tissue Freezing Medium (Electron Microscopy Services, Hatfield, PA) using 2-Methylbutane (Sigma, St. Louis, MO) cooled by liquid nitrogen. 12 **μ**m thick sections were cut on a Lieca CM 3050 S cryostat and stored at -20°C prior to use. Immediately before use, frozen sections were treated with 4% paraformaldehyde in PBS for 30 min. Both paraffin and frozen sections were brought to ambient temperature and treated with blocking solution (4% non-immune goat serum, 0.1% Triton X-100, 0.05% SDS, and 0.1% fish skin gelatin [Sigma] in TBST [0.05% Tween-20 in Tris-buffered saline, pH 7.4]) for 30 min, subsequently washed with TBST and then exposed to primary antibodies overnight at 4°C. Next day the sections were washed with TBST, incubated with Alexa Fluor-conjugated secondary antibodies (Life Technologies, Grand Island, NY) for 30 min at 22**°**C, and washed with TBST followed by a rinse with TBS. The antibodies were brought to their working dilutions with 0.1% fish skin gelatin in TBS. The sections were then dipped for 5 s in DAPI (1 μg/ml in TBS) and after rinsing with TBS were mounted with Prolong Gold (Life Technologies). Each kidney image is a maximum projection of 16 confocal images taken at 0.5 μm intervals.

Mouse posterior eyecups were fixed for 1 hr with 4% paraformaldehyde in mouse Ringer’s solution, then rinsed three times in Ringer’s. Fixed eyecups were embedded in Tissue-Tek O.C.T. (Sakura, Torrance, CA) and flash frozen on liquid nitrogen for cryostat sectioning. 18 **μ**m thick sections were cut on a Microm HM550 cryostat (Thermo Fisher Scientific, Grand Island, NY) and stored at -20**°**C prior to use. Immediately before use, frozen sections were brought to ambient temperature and incubated in blocking solution (5% goat serum and 0.5% Triton X-100 in PBS) for 1 hr. Sections were incubated in primary antibodies in blocking solution overnight at 4**°**C, rinsed three times, and incubated with goat secondary antibodies conjugated with Alexa Fluor 488, 568, or 647 (Invitrogen) in blocking solution for 2 hr at 22**°**C. To stain nuclei, 5 **μ**g/ml Hoechst (Invitrogen) was used. To stain retinal layers, 1 **μ**g/ml lectin Wheat Germ Agglutinin (WGA) conjugated to Alexa594 (Thermo Fisher Scientific) was used. Sections were mounted with Immu-Mount (Thermo Fisher Scientific) and cover-slipped. Images were acquired using a Nikon Eclipse 90i microscope and a C1 confocal scanner controlled by EZ-C1, version 3.10 software.

#### Antibodies

Aquaporin-2, rabbit polyclonal (Sigma); Gamma tubulin, mouse monoclonal clone GTU-88 (Sigma); Arl13b, mouse monoclonal clone N295B/66 (NeuroMab, University of California Davis); T1α, hamster monoclonal clone 8.1.1 (Developmental Studies Hybridoma Bank, University of Iowa); Perilipin-A, goat polyclonal (Abcam, Cambridge, MA); Insulin, guinea pig polyclonal (DAKO); Glucagon, mouse monoclonal clone K79bB10 (Sigma); *Helix pomatia* agglutinin, Alexa488 conjugated (Thermo Fisher Scientific); Lotus Tetragonolobus Agglutinin, fluorescein labeled (Vector Labs, Burlingame, CA); Arf4, rabbit polyclonal antibody against residues 98–114 of mouse Arf4; Rhodopsin, monoclonal clone 1D4 (Abcam); Peripherin, rabbit polyclonal antibody (Gabriel Travis, University of California Los Angeles); Phosducin, mouse monoclonal clone G-7 (Santa Cruz, Dallas, TX); GM130, mouse monoclonal clone 35 (BD Biosciences, San Jose, CA); B-actin, mouse monoclonal clone C4 (Santa Cruz); Hsp90, rabbit polyclonal (Santa Cruz).

### Western blotting

Tissues from experimental and control mice were collected, homogenized and sonicated in 2% sodium dodecyl sulfate (Sigma) and 1× cOmplete protease inhibitor mixture (Roche, Indianapolis, IN) in phosphate-buffered saline (PBS). Eyecup lysates were cleared at 14,000 g for 10 min at 22°C and total protein concentration was measured using the RC DC Protein Assay kit (Bio-Rad, Hercules, CA). Kidney and pancreas lysates were cleared at 100,000 g for 20 min at 22°C and total protein concentration was measured using the BCA Protein Assay Kit (Pierce). Serial dilutions of each lysate were subjected to SDS polyacrylamide gel electrophoresis using a 10–20% Tis-HCL criterion gel (Bio-Rad) and transferred to Immun-Blot LF PVDF (Bio-Rad). Western blotting was performed by incubating in the appropriate primary antibody diluted in 50% / 50% of Odyssey blocking buffer / PBST overnight at 4°C. Blots were then rinsed 3 times with PBST before incubating in the corresponding secondary goat antibodies conjugated with Alexa Fluor 680 or 800 (Invitrogen) for 2 hr, at 22°C. Bands were visualized and quantified using the Odyssey infrared imaging system (LiCor Bioscience, Lincoln, NE).

## Supporting information

S1 Fig No evidence for general microvilli dysfunction. **A.** Transmission electron micrographs of small intestine (SI) from control and experimental *Arf4*^*flox*^*/CagCreER* mice. Microvilli uniformly covered the cells from both mice and appeared structurally normal. Scale bar = 1 μm. **B.** Transmission electron micrographs of kidney proximal tubules from control and experimental *Arf4*^*flox*^*/CagCreER* mice. Tubule cells and their associated microvilli were not detectably different between the two genotypes. Scale bar = 2 μm.
(TIF)

S2 Fig Western blot from *Arf4*^*flox*^*/CagCreER* experimental and control eyecup lysates show the presence of nonspecific bands upon staining with the anti-Arf4 antibody. 20 μg of total protein extract from control and experimental mouse eyecups is loaded in each lane.
(TIF)

S3 Fig Quantitative western blot analysis of Arf4 reduction in tamoxifen treated *Arf4*^*flox*^*/CagCreER* kidney and in Arf4flox/HoxB7-Cre kidney papilla. **A**. Representative Western blots show serial dilutions of control (*Arf4*^*+/flox*^*/CagCreER*) and experimental (*Arf4*^*flox/flox*^*/CagCreER*) kidney lysates for Arf4 and Hsp90 proteins. The fluorescent signal produced by the Arf4 band was normalized to Hsp90 and plotted versus total protein loaded. The slope of the curves was used to calculate the amount of each protein in control and experimental kidneys. **B**. Representative Western blots show serial dilutions of control (*Arf4*^*+/flox*^*/HoxB7-Cre*) and experimental (*Arf4*^*flox/flox*^*/HoxB7-Cre*) kidney papilla lysates for Arf4 and Hsp90 proteins. The fluorescent signal produced by the Arf4 band was normalized to Hsp90 and plotted versus total protein loaded. The slope of the curves was used to calculate the amount of each protein in control and experimental papilla.
(TIF)

S4 Fig Quantitative western blot analysis of Arf4 reduction in tamoxifen treated *Arf4*^*flox*^*/CagCreER* pancreas. **A.** P10 representative Western blots show serial dilutions of control (*Arf4*^*+/flox*^*/CagCreER*) and experimental (*Arf4*^*flox/flox*^*/CagCreER*) pancreas lysates for Arf4 and Hsp90 proteins. The fluorescent signal produced by the Arf4 band was normalized to Hsp90 and plotted versus total protein loaded. The slope of the curves was used to calculate the amount of each protein in control and experimental pancreas. **B.** P28 representative Western blots show serial dilutions of control (*Arf4*^*+/flox*^*/CagCreER*) and experimental (*Arf4*^*flox/flox*^*/CagCreER*) pancreas lysates for Arf4 and Hsp90 proteins. The fluorescent signal produced by the Arf4 band was normalized to Hsp90 and plotted versus total protein loaded. The slope of the curves was used to calculate the amount of each protein in control and experimental pancreas.
(TIF)
