# Supplementary figures and images for: Loss of Arf4 causes severe degeneration of the exocrine pancreas but not cystic kidney disease or retinal degeneration
Source: PLoS Genet. 2017 Apr 14;13(4):e1006740. doi: 10.1371/journal.pgen.1006740 (PMC5409180; doi:10.1371/journal.pgen.1006740)

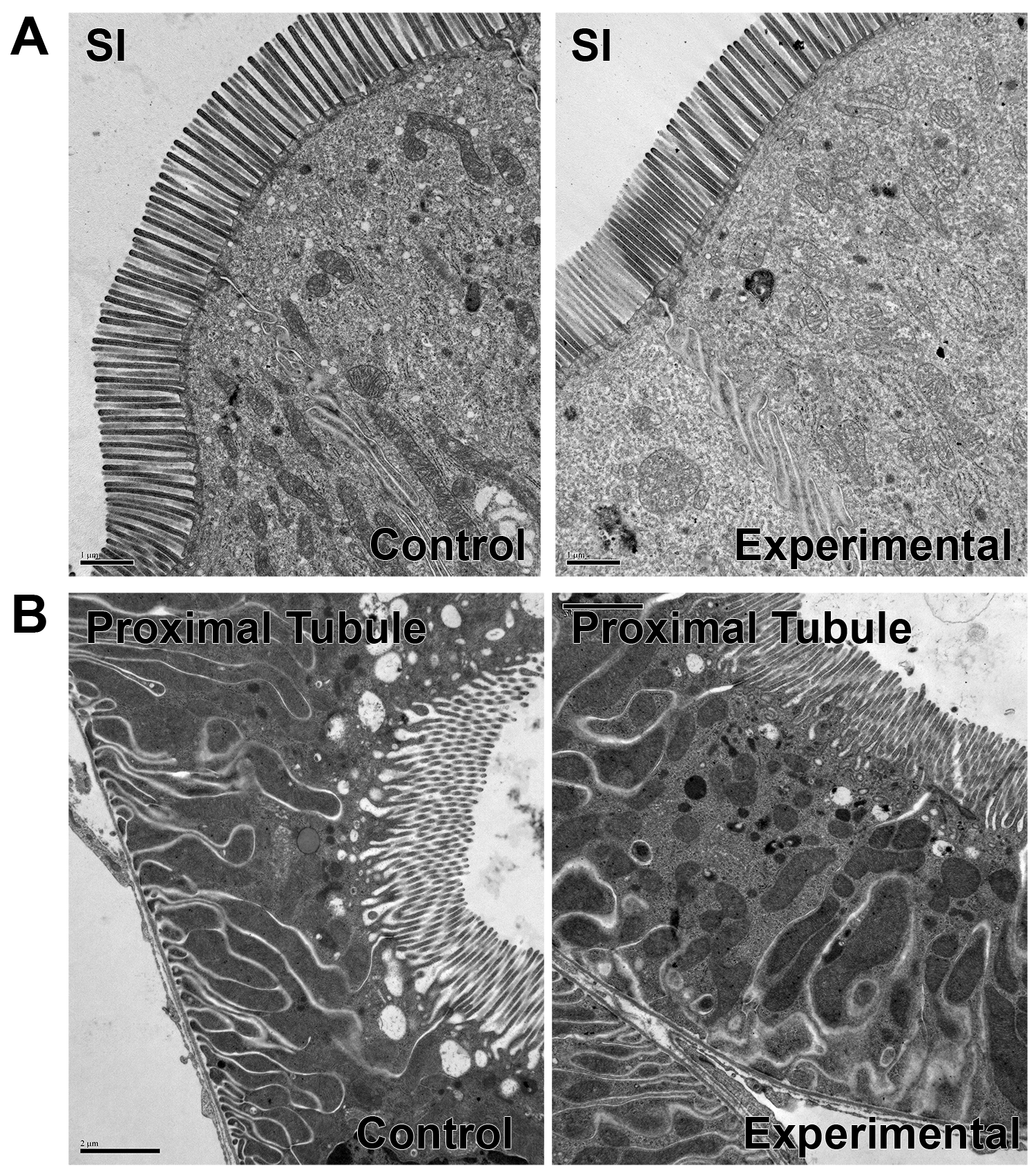

Supplement: S1 Fig — A. Transmission electron micrographs of small intestine (SI) from control and experimental Arf4flox/CagCreER mice. Microvilli uniformly covered the cells from both mice and appeared structurally normal. Scale bar = 1 μm. B. Transmission electron micrographs of kidney proximal tubules from control and experimental Arf4flox/CagCreER mice. Tubule cells and their associated microvilli were not detectably different between the two genotypes. Scale bar = 2 μm. (TIF) [file pgen.1006740.s001.tif]

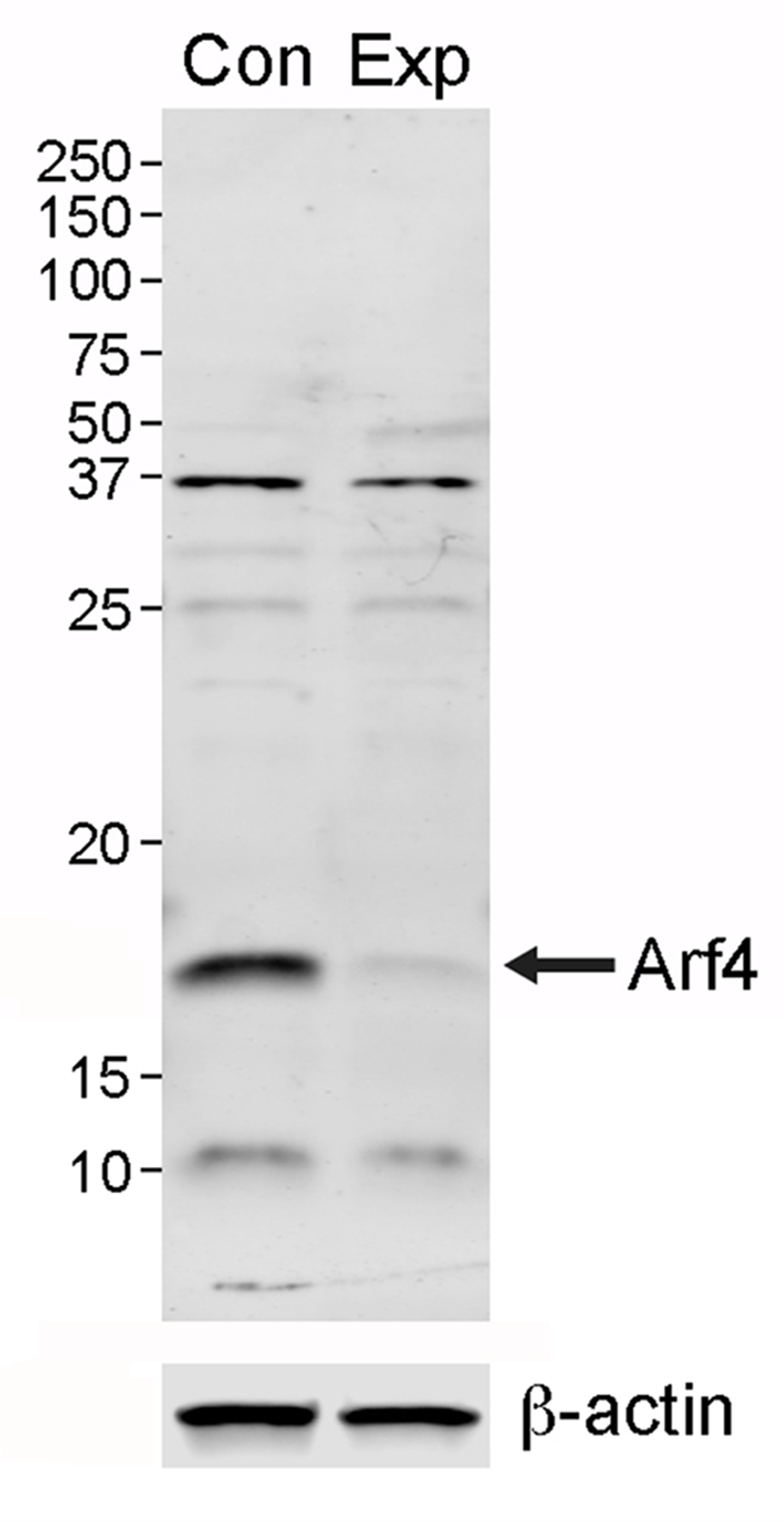

Supplement: S2 Fig — 20 μg of total protein extract from control and experimental mouse eyecups is loaded in each lane. (TIF) [file pgen.1006740.s002.tif]

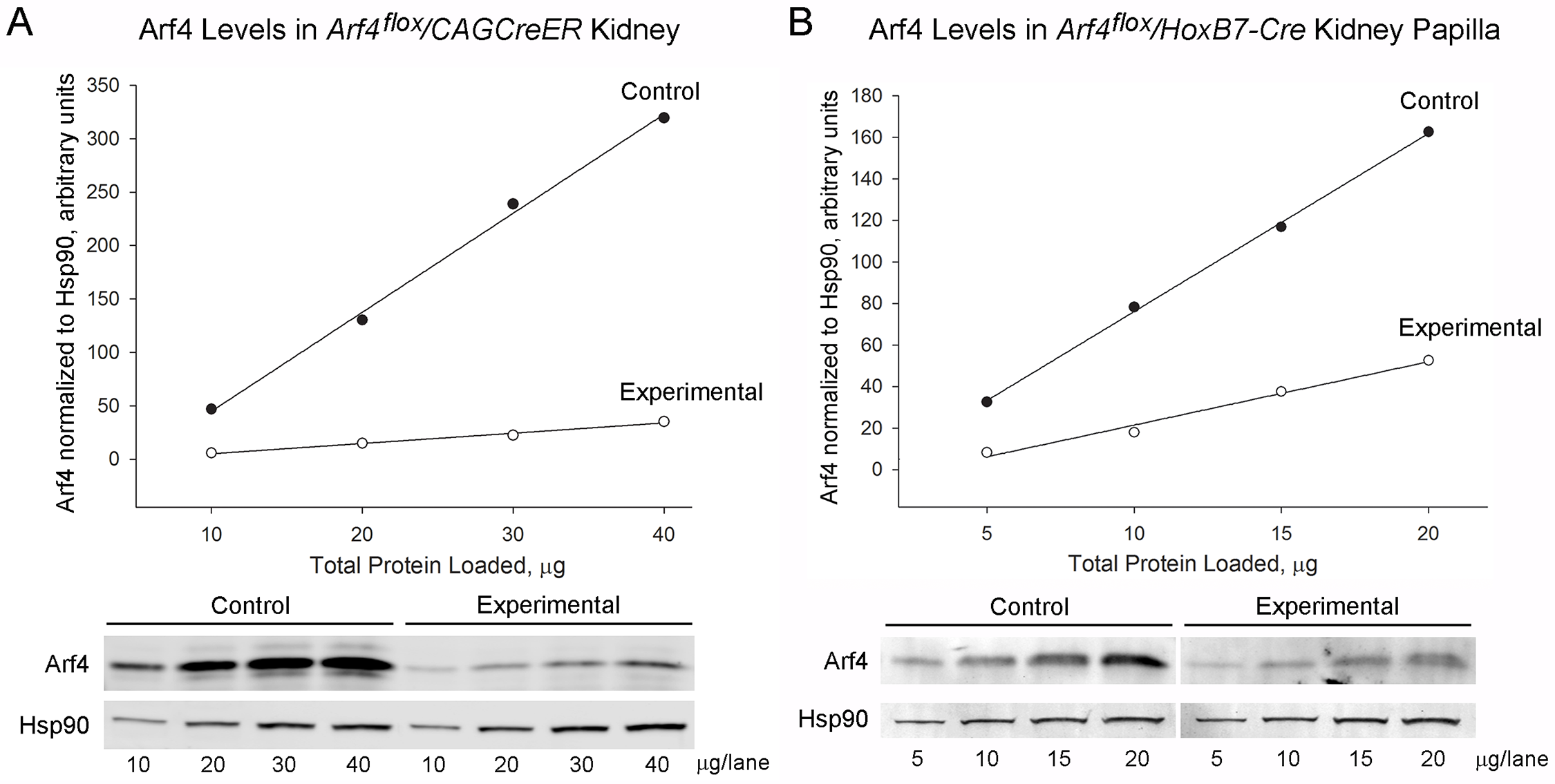

Supplement: S3 Fig — A. Representative Western blots show serial dilutions of control (Arf4+/flox/CagCreER) and experimental (Arf4flox/flox/CagCreER) kidney lysates for Arf4 and Hsp90 proteins. The fluorescent signal produced by the Arf4 band was normalized to Hsp90 and plotted versus total protein loaded. The slope of the curves was used to calculate the amount of each protein in control and experimental kidneys. B. Representative Western blots show serial dilutions of control (Arf4+/flox/HoxB7-Cre) and experimental (Arf4flox/flox/HoxB7-Cre) kidney papilla lysates for Arf4 and Hsp90 proteins. The fluorescent signal produced by the Arf4 band was normalized to Hsp90 and plotted versus total protein loaded. The slope of the curves was used to calculate the amount of each protein in control and experimental papilla. (TIF) [file pgen.1006740.s003.tif]

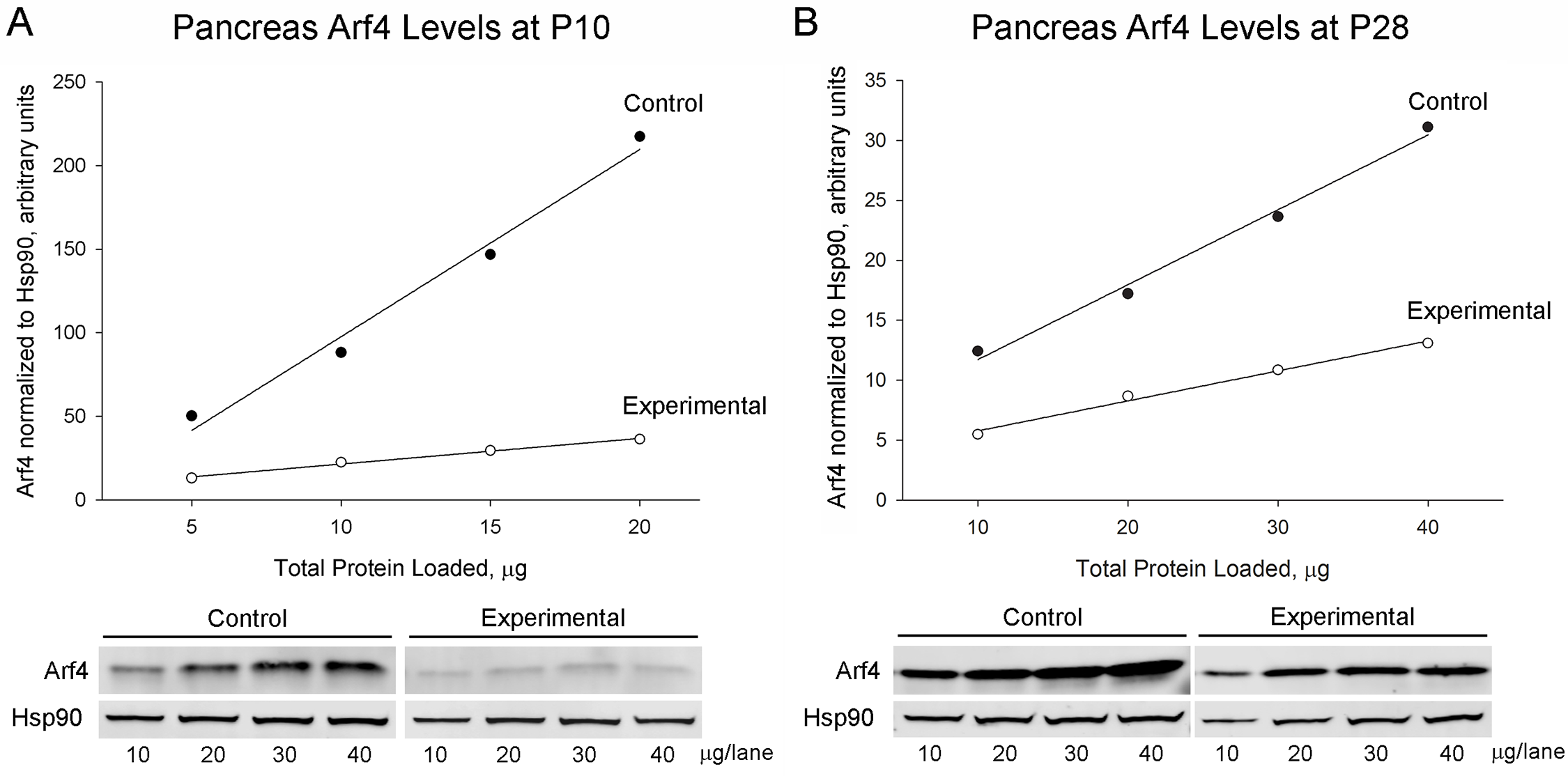

Supplement: S4 Fig — A. P10 representative Western blots show serial dilutions of control (Arf4+/flox/CagCreER) and experimental (Arf4flox/flox/CagCreER) pancreas lysates for Arf4 and Hsp90 proteins. The fluorescent signal produced by the Arf4 band was normalized to Hsp90 and plotted versus total protein loaded. The slope of the curves was used to calculate the amount of each protein in control and experimental pancreas. B. P28 representative Western blots show serial dilutions of control (Arf4+/flox/CagCreER) and experimental (Arf4flox/flox/CagCreER) pancreas lysates for Arf4 and Hsp90 proteins. The fluorescent signal produced by the Arf4 band was normalized to Hsp90 and plotted versus total protein loaded. The slope of the curves was used to calculate the amount of each protein in control and experimental pancreas. (TIF) [file pgen.1006740.s004.tif]
